# A Genome-Scale Insight into the Effect of Shear Stress During the Fed-Batch Production of Clavulanic Acid by *Streptomyces Clavuligerus*

**DOI:** 10.3390/microorganisms8091255

**Published:** 2020-08-19

**Authors:** David Gómez-Ríos, Victor A. López-Agudelo, Howard Ramírez-Malule, Peter Neubauer, Stefan Junne, Silvia Ochoa, Rigoberto Ríos-Estepa

**Affiliations:** 1 Grupo de Investigación en Simulación, Diseño, Control y Optimización de Procesos (SIDCOP), Departamento de Ingeniería Química, Universidad de Antioquia UdeA, Calle 70 No. 52-21, Medellín 050010, Colombia; silvia.ochoa@udea.edu.co; 2 Grupo de Bioprocesos, Departamento de Ingeniería Química, Universidad de Antioquia (UdeA), Calle 70 No. 52-21, Medellín 050010, Colombia; valonso.lopez@udea.edu.co; 3 Escuela de Ingeniería Química, Universidad del Valle, A.A. 25360, Cali 76001, Colombia; howard.ramirez@correounivalle.edu.co; 4 Technische Universität Berlin, Institute of Biotechnology, Chair of Bioprocess Engineering, Ackerstr. 76, ACK 24, D-13355 Berlin, Germany; peter.neubauer@tu-berlin.de (P.N.); stefan.junne@tu-berlin.de (S.J.); 5 Escuela de Biociencias, Universidad Nacional de Colombia sede Medellín, Calle 59 A 63-20, Medellín 050010, Colombia

**Keywords:** *Streptomyces*, genome-scale model, flux balance analysis, clavulanic acid, *Streptomyces clavuligerus*, TCA cycle, secondary metabolism, metabolic modeling, antibiotic

## Abstract

*Streptomyces clavuligerus* is a filamentous Gram-positive bacterial producer of the β-lactamase inhibitor clavulanic acid. Antibiotics biosynthesis in the *Streptomyces* genus is usually triggered by nutritional and environmental perturbations. In this work, a new genome scale metabolic network of *Streptomyces clavuligerus* was reconstructed and used to study the experimentally observed effect of oxygen and phosphate concentrations on clavulanic acid biosynthesis under high and low shear stress. A flux balance analysis based on experimental evidence revealed that clavulanic acid biosynthetic reaction fluxes are favored in conditions of phosphate limitation, and this is correlated with enhanced activity of central and amino acid metabolism, as well as with enhanced oxygen uptake. In silico and experimental results show a possible slowing down of tricarboxylic acid (TCA) due to reduced oxygen availability in low shear stress conditions. In contrast, high shear stress conditions are connected with high intracellular oxygen availability favoring TCA activity, precursors availability and clavulanic acid (CA) production.

## 1. Introduction

*Streptomyces clavuligerus* (*S. clavuligerus*) is a biotechnologically important Gram-positive filamentous bacterium for the production of β-lactam antibiotic compounds such as clavulanic acid (CA) and cephamycin C. In the current emergence of extended multidrug and antibiotic resistance phenomena, the β-lactam inhibitors remain as one of the main drugs used against bacterial resistant infections [1]. CA has a potent inhibitory activity on β-lactamase enzymes responsible for bacterial resistance to several β-lactam antibiotics of extended clinical use. CA is usually produced in submerged cultures under appropriate nutritional conditions. However, CA is unstable in aqueous solutions including fermentation broths and its productivity is compromised in both production and downstream processes [2,3]. Several studies have focused on assessing the effect of nutrients and environmental conditions on CA production and have identified a variety of operational conditions that presumably favor its accumulation during the cultivation process, as previously reviewed by Ser et al. [4]. It is likely that phosphate limitation and oxygen supply play an important role in the activation of secondary metabolism and CA biosynthesis in *S. clavuligerus* [5,6]. Nevertheless, the metabolic response of *S. clavuligerus* to different environmental and nutritional factors and its relationship with CA biosynthesis is not completely understood. In this regard, a strong correlation between macromorphology and CA production under low and high shear stress conditions in submerged cultures of *S. clavuligerus* was recently reported [7]. The study describes that high shear forces, as they occur in stirred tank bioreactors at typical stirrer speeds for sufficient oxygen supply at elevated biomass concentrations, do not compromise the viability of *S. clavuligerus* cells, as comparably high specific growth and CA production rates were achieved. Additionally, it was assumed that CA production might be affected by a lower surface-to-volume ratio at thicker mycelia evolving under low-shear cultivation conditions, which would impact negatively upon the diffusion capacity and, therefore, the transport of nutrients, oxygen and product secretion.

CA is a product of the so-called clavam pathway. The clavam pathway is usually divided into the early steps, including the reactions from *N*_2_-(2-carboxy-ethyl) L-arginine to (*3S, 5S*)-clavaminic acid, and the late steps, comprising the reactions up to CA and the clavam 5S compounds [8,9]. The first reaction of the pathway involves a condensation reaction between L-arginine and glyceraldehyde-3-phosphate to produce N_2_-(2-carboxy-ethyl) L-arginine in a reaction catalyzed by the N_2_-(2-carboxyethyl) arginine synthase (CEAS). Glycerol is the preferred substrate for production of CA because of its rather direct conversion into glyceraldehyde 3-phosphate (GAP). GAP can be metabolized through three different pathways: (i) the gluconeogenesis and pentose phosphate pathway, (ii) the glycolysis and tricarboxylic acid (TCA) cycle and (iii) the clavam and CA biosynthesis pathway [10].

**Figure 1:**
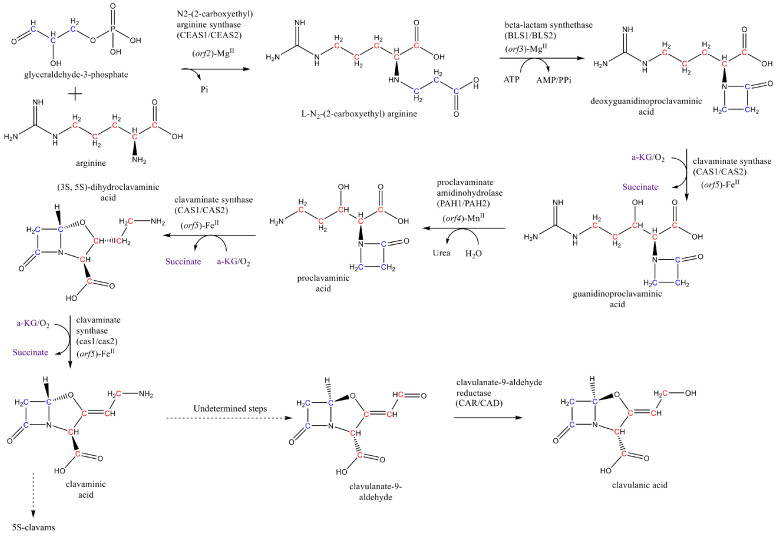
Clavulanic acid biosynthesis pathway.

Subsequently, deoxyguanidinoproclavaminic acid, a β-lactam compound, is formed by intramolecular reaction of *N*2-(2-carboxy-ethyl) L-arginine by action of β-lactam synthetase (BLS) [11,12]. Enzyme clavaminate synthase (CAS) is a 2-oxoglutarate (α-ketogutarate) dependent oxygenase catalyzing three reaction of the clavam pathway, the first of them being the hydroxylation of deoxyguanidinoproclavaminic acid forming guanidinoproclavaminic acid [12,13]. The amidino group in the residue of arginine in guanidinoproclavaminic acid is removed by action of proclavaminate amidino hydrolase (PAH), producing proclavaminic acid [14,15]. Proclavaminic acid forms dihydroclavaminic acid following an oxidative cyclization mechanism in the second reaction catalyzed by CAS, which is followed by an oxidative desaturation catalyzed by CAS, yielding the (3S, 5S)-clavaminic acid [12,16]. At this point, the clavam pathway bifurcates into two branches, one leading to CA having clavulanate-9-aldehyde as intermediate, and the other producing several clavam 5S compounds. Notice that the 3S, 5S stereochemistry of clavaminic acid is conserved in the synthesis of clavams 5S; nevertheless, stereochemical inversion is required in the late steps leading to CA. Some authors suggested that N-glycyl-clavaminic acid might be an intermediate in the late steps of CA biosynthesis, which also would have a key role in the stereochemical inversion of 3S, 5S configuration into 3R, 5R of clavulanate-9-aldehyde and CA [17]. Clavulanate-9-aldehyde is finally reduced by the clavulanate dehydrogenase (CAD) into CA [18]. The three reactions catalyzed by CAS are rate-limiting steps and control the flux reaction along the clavulanic acid and 5S clavams pathways [19,20]. CA biosynthesis pathway is summarized in Figure 1.

A common approach for studying the effect of nutritional, genetic and environmental perturbations on metabolism is the genome-scale metabolic modeling using flux balance analysis (FBA) [21]. In the case of *S. clavuligerus*, metabolic network models have been reconstructed and/or updated for this purpose [10,22,23,24], providing insights about the metabolic features of the species and possible genetic targets for further strain improvement. Nevertheless, the referred models share a common origin and some of their inconsistencies persisted between them without being corrected.

Starting from recent advances in genome-scale model reconstruction and available genetic information, a new and improved genome-scale model of the *S. clavuligerus* metabolism has been developed in this work. A novel top-down automated reconstruction tool proposed by Machado et al. [25] was used for the generation of an initial model based on the genome assembly for *S. clavuligerus* by Cao et al. [26]. The initial reconstruction was systematically and manually curated for inclusion of missing reactions and the removal of inconsistencies, especially those of thermodynamic nature, for the purpose of obtaining a more realistic representation of the complexity of the *S. clavuligerus* metabolism. This model was used in a combined approach of experimental and modeling work for investigating the connection between the different nutritional conditions observed in *S. clavuligerus* cultivations and CA biosynthesis. Defined media was used to study the influence of nutrients on carbon flux distribution during CA biosynthesis [10,27]. Previous studies also have suggested that amino acid supplementation could affect the specialized metabolite accumulation in *S. clavuligerus* given their connections with amino acid biosynthesis and degradation pathways [27,28]. In this case a defined medium was used for fed-batch cultivations of *S. clavuligerus* in a stirred tank and single-use 2-D rocking-motion bioreactors. A medium rich in glycerol but limited in phosphate and glutamate was used as feed in order to explore the effects of their depletion on the CA biosynthesis rate. Subsequently, the experimentally observed metabolic scenarios were simulated using the constructed and validated genome-scale model of *S. clavuligerus* in order to identify putative connections between the central carbon and amino acid metabolism, nutrient limiting conditions and CA production.

## 2. Materials and Methods

### 2.1. Generation of a New Genome Scale Model of S. Clavuligerus

*S. clavuligerus* representative genomes range between 7.6 and 8.5 Mb with a GC content of 72.6% and a median protein count of 6654. An initial Genome-Scale Metabolic Model (GEM) was generated from the representative genome of *S. clavuligerus,* sequenced by Cao et al. [26] and currently available in the database of the National Center for Biotechnology Information (Bethesda, MD, USA) with the refseq identifier GCA_001693675.1. For this initial reconstruction the automated tool for GEM reconstruction CarveMe was used [25]. The reconstruction pipe-line of this novel tool is defined as a top-down approach, in which the probable reactions of the organism-specific metabolic network are scored according to the Gene-Protein-Reaction (GPR) associations from the organism genome assembly [25]. For the initial reconstruction of the GEM of *S. clavuligerus*, the script of CarveMe was executed in the Anaconda Python distribution for Linux; externally, the Diamond sequence aligner [29] and IBM CPLEX v12.9 for Linux were also required.

The initial GEM was manually curated by following a systematic procedure in order to improve the in silico representation of the physiology of the organism. The missing reactions and metabolites in the secondary metabolism, which correspond to penicillin–cephalosporin biosynthesis, clavulanic acid biosynthesis and 5S-clavams bifurcation were manually added and associated to the corresponding genes, while maintaining the connectivity of the model, and the mass and charge balances, respectively.

The flux distribution, obtained by solving a standard FBA optimization, frequently include thermodynamic infeasible cycles (TICs) that represent slopes of reactions like a perpetual motion machine that violates the Second Law of Thermodynamics. This heavily distorts the reaction flux predictions. It has been demonstrated that the number of infeasible loops grows rapidly with the network size [30]. Therefore, the construction of larger metabolic models usually implies a considerable number of TICs. Some FBA strategies are currently available for avoiding the TICs, e.g., the thermodynamic FBA that imposes an extra set of constraints for ∆Gr and reaction directionality if necessary [31,32]. The loopless FBA and CycleFreeFlux approaches do not require extra parameters in the model [30,33]. Nevertheless, these might not eliminate all TICs in the network, although it can improve the FBA predictions [32]. In this work a Matlab^®^ implementation of the Flux Variability Analysis (FVA) methodology proposed by Schellenberger et al. [30] for TICs identification was used in order to determine reactions that appear in one or more TICs. Within this methodology, the TICs are a set of reactions linked to unbounded metabolic fluxes with no specificity for substrate uptake when applying FVA. For the FVA, the substrate uptakes were constrained to 1.0 mmol. (g DCW.h)^−1^.

The set of unbounded reactions which are part of the TICs were used to define a stoichiometric matrix. Its null space was used for the individual identification of TICs composed by two or more reactions. The manual curation of the TICs was performed by (i) elimination of linearly dependent reversible reactions resulting in redundancy due to erroneous identification of GPR during the automated reconstruction, and (ii) restriction of the reaction directionality by calculation of the correspondent Standard Gibbs free energy of reaction (∆Gr) using the NExT (Network-Embedded Thermodynamic analysis) algorithm [34]. The scripts implemented for TIC identification are available in Supplementary Material S1.

The final GEM, denoted as iDG1237, was reviewed with the MC3 Consistency Checker algorithm, which implements stoichiometric-based identification and FVA analysis to determine single connected and dead end metabolites in the network, as well as the consistent coupled and inconsistent coupled/zero-flux reactions [35]. The iDG1237 genome-scale model for *S. clavuligerus* is available in SBML (xml) and MS Excel (xlsx) file formats in the Supplementary Material S2, including the Memote consistency report [36].

### 2.2. Flux Balance Analysis

In the FBA studies the external/internal exchange reactions were constrained to represent, in silico, the environmental conditions observed in *S. clavuligerus* cultivations at high and low shear stress conditions. FBA solves a linear programming problem for calculating the steady-state reaction flux distributions along the metabolic network that maximizes (or minimizes) a pre-defined objective function, under specific constraints. The objective function shall reflect the achievement of a meaningful physiological state of the organism like a maximal ATP production, growth rate, or specific metabolite production [37]. Some variations of the traditional FBA, such as the parsimonious and sparse FBA, use a two-stage optimization procedure in order to retrieve a unique and biologically meaningful solution to the FBA problem of growth rate maximization. In this work a two-stage approach involving biomass maximization and FBA and flux minimization was used, as presented in Equation (1).

(1) $$Min\;\sum v^{2}$$

(2) $$\left[\begin{array}{c} s.t.\;Sv=0\;\\ \;l_{b}\leq v\leq l_{u}\\ \;f^{T}v=Z_{max} \end{array}\right]$$

where ***S*** is the stoichiometric matrix of coefficients for *m* metabolites and *n* reactions, *v* is the flux vector of dimension *n*, *Z_max_* represents the biomass maximization, *f* is a vector of weights, and *l_b_* and *u_b_* are the lower and upper bounds, respectively. Thus, in the first stage the FBA standard problem for biomass maximization is solved, whereas the second stage deals with the minimization of the vector of fluxes. It is important to notice that the assumption underlying the objective function minimization, postulates that living organisms gain functional fitness by fulfilling their functions with maximal efficiency, and thus assuring a minimal energy requirement to accomplish a specific pattern of cellular functions [38]. All FBA problems were solved in COBRA Toolbox 3.0 in MATLAB R2018a; the Gurobi 7.0 and IBM CPLEX v12.9 optimization plugins were used for the solution of two-steps FBA and FVA, respectively.

### 2.3. Microorganism, Cultivation Media and Experimental Conditions

*S. clavuligerus* DSM 41826, cryo-preserved at −80 °C in glycerol solution (16.7% v/v), was inoculated for activation in seed medium as described by Roubos et al. [39]. Cultivations were carried out in duplicate in a 15 L stirred tank bioreactor (Techfors S, Infors AG, Bottmingen, Switzerland) and a 20 L single-use 2-D rocking-motion bioreactor CELL-tainer^®^ (Cell-tainer Biotech BV, Winterswijk, The Netherlands) both operating at 5 L initial filling volume. Chemically defined media were used in all cultivations, prepared as follows (in g.L^−1^ deionized and distilled water): glycerol (9.3), K_2_HPO_4_ (0.8), (NH_4_)_2_SO_4_ (1.26), monosodium glutamate (9.8), FeSO_4_·7H_2_O (0.18), MgSO_4_·7H_2_O (0.72) and trace element solution (1.44 mL). The trace elements solution contained (in g.L^−1^ deionized and distilled water): H_2_SO_4_ (20.4), monosodium citrate·1H_2_O (50), ZnSO_4_·7H_2_O (16.75), CuSO_4_·5H_2_O (2.5), MnCl_2_·4H_2_O (1.5), H_3_BO_3_ (2), and Na_2_MoO_4_·2H_2_O (2) (all reactants from Carl Roth GmbH, Karlsruhe, Germany). Antifoam 204 (Sigma Inc., St. Louis, MO, USA) was used at concentration of 1:1000 v/v. Bioreactors were inoculated (10% v/v) and operated at 28 °C; aeration at 0.6 VVM was provided and pH was maintained at 6.8 by addition of NaOH 3M and HCl 3M(Carl Roth GmbH, Karlsruhe, Germany). Reactors were equipped with pH (Polylite Plus) dissolved oxygen (DO–VisiFerm) probes (Hamilton Inc., Bonaduz, Switzerland), O_2_, CO_2_ gas sensors and exhaust gas analyzer (BlueInOne Ferm, BlueSens GmbH, Herten, Germany).

It has been reported that *S. clavuligerus* adapts differently to motion patterns and shear forces [7]. Therefore, cultivations were performed in both reactors maintaining similar DO levels by controlling the agitation velocity. Batch cultivation was performed during the first 37 h until glycerol and phosphate were depleted. Then, the fed-batch operation started at a constant rate of 35 mL.h^−1^ with a defined medium prepared as follows (in g.L^−1^ deionized and distilled water): glycerol (120.0), K_2_HPO_4_ (2.0), (NH_4_)_2_SO_4_ (8.0), until a final volume of 7.8 L was reached. During the batch operation DO was not controlled, only minimum DO was set at 20% to avoid anoxia. During fed-batch operation DO was maintained rather constant at 62 ± 5%. Culture samples (2 mL) were taken at 12 h intervals and centrifuged at 15,000 rpm and 4 °C for 10 min; wet biomass was washed with 0.9% NaCl, centrifuged and dried overnight at 75 °C for dry cell weight (DCW) determination.

CA concentration in supernatant samples was determined in a HPLC (1200 Series, Agilent Technologies GmbH, Waldbronn, Germany) with a diode array detector (DAD), using a Zorbax Eclipse XDB-C-18 chromatographic column (Agilent Technologies) and a C-18 guard column (Phenomenex^®^ GmbH, Aschaffenburg, Germany) operated with a flow rate of 1 mL.min^−1^ at 30 °C. The mobile phase consisted of H_2_PO4 (50 mM, pH 3.2) and methanol (HPLC grade). Analyses were performed using the gradient method described by Ramirez-Malule [40]. Imidazole was used for derivatization of CA; the clavulanate–imidazole chromophore was detected at 311 nm.

Glycerol, glycolysis and TCA intermediates were quantified in a HPLC (1200 series, Agilent Technologies) with a refractive index detector (RID) and operated at 15 °C using a HyperREZ™ XP carbohydrate H+ column (Thermo Scientific, Waltham, MA, USA) at a constant flow rate of 0.5 mL.min^−1^ using 5 mM sulfuric acid solution as mobile phase [41]. Quantifications of amino acids were performed in a HPLC (Agilent 1260 Series Infinity, Agilent Technologies) with a fluorescence detector (FLD) at an excitation wavelength of 340 nm and an emission wavelength of 450 nm. o-Phthaldialdehyde was used for precolumn derivatization of the samples. A C18 Gemini^®^ column with a SecurityGuard™ precolumn (Phenomenex) were used, and operated at a flow rate of 1 mL.min^−1^ and 40 °C. Mobile phase consisted of NaH_2_PO_4_ (40 mM, pH 7.8) as polar eluent and a solution of methanol (45% vol.), acetonitrile (45% vol.) and water (10% vol.) as nonpolar eluent [42]. Semi-quantitative determination of phosphate and ammonium ions was performed by using phosphate and ammonia tests (MQuant™; Merck KGaA, Darmstadt, Germany).

## 3. Results and Discussion

### 3.1. Development of a New and Improved Genome Scale Model of S. Clavuligerus

Firstly, the aim of this study was to develop a novel GEM for *S. clavuligerus* in order to improve the in silico representation of its metabolism in comparison to previously reported GEMs. It considers the current state-of-the-art in genetics and biochemistry. The reconstruction process was based on an initial functional and high-quality universal model, in which infeasible reactions for *S. clavuligerus* were removed and constraints were added [25]. However, this organism-specific model reconstruction presented numerous gaps in pathways and gene annotations in addition to a considerable number of TICs. The initial reconstruction included 1956 metabolic reactions, 233 internal/external exchange reactions and 1237 GPR associations. After a preliminary manual curation of reactions, the model considered 2004 metabolic reactions, 243 internal/external exchange reactions and 1257 GPRs.

The previous GEM share a common origin, the model iMM865 reported by Medema et al. [43]. The derived models have lower numbers of genes, metabolites and reactions than the new reconstruction, as presented in Table 1. After a first manual curation, a total of 1018 unbounded reactions taking part in 124 TICs were identified and reactions participating in infeasible loops were reviewed for linear independence, directionality and essentiality for growth. The manual curation of TICs allowed the elimination of 70 linearly dependent reactions associated to cofactors and prosthetic group biosynthesis, alternate carbon metabolism, amino acid metabolism, membrane lipid metabolism and erroneous inner membrane transport reactions. Additionally, the directionality of 20 reactions was restricted according to the calculated ∆Gr. After implementing these changes, the number of unbounded reactions was reduced to 86 and the TICs were curated in 93% of reactions. The remaining loops could not be removed due to their intrinsic connectivity with essential reactions in the network.

The resulting GEM of *S. clavuligerus* named iDG1237 consisted of 2177 reactions, including 543 internal/external metabolite exchange reactions and 1237 annotated genes. The metabolites were annotated according with their correspondent BiGG identifiers; similarly, the genes were annotated with their Refseq identifiers when available. The model consistency statistics for the iDG1237 model and the previous models for *S. clavuligerus* are presented in Table 1.

The improved connectivity of the model is reflected in a lower number of dead-end metabolites. In the case of the iDG1237 GEM, the number of dead-end metabolites is considerably lower (3%) compared to previously reported GEMs (~27%). Additionally, the number of zero-flux reactions and inconsistent coupling was approximately 80% lower than in previous reconstructions. Therefore, the full consistent coupling allows better determination of relationships among reactions and pathways, connections between nutritional factors and desired metabolites, and analysis of potential knockouts, as well as further regulatory and expression studies [35,44]. The number of infeasible loops is also considerably lower in the iDG1237 model and therefore, the loopless condition in FBA simulations might be easily satisfied, leading to more consistent metabolic phenotype predictions. In summary, the iDG1237 model presents fewer inconsistencies in the context of pseudo steady-state modelling than the previous models reported for *S. clavuligerus*.

### 3.2. Model Validation

The model validation was performed by comparing the fluxes predicted by the iDG1237 model and the previous GEMs against experimental data of *S. clavuligerus* in a chemostat mode, using the experimental data published by Bushell et al. [27] and Ramirez-Malule et al. [10]. Simulations were performed under the two-stage optimization approach. The experimental specific growth rate in chemostat cultures was compared with model predictions. Exchange fluxes of glycerol (*v_ex,Glyc_*), glutamate (*v_ex,Glu_*), and phosphate (*v_ex,Pi_*) were constrained according to experimental data [10,27]. The exchange reactions bounds for other media components and oxygen were set to allow free import. The values of the experimental (*μ_exp_*) and in silico (*μ*) growth rates, CA secretion fluxes (*v_ex,CA_*) and cumulative mean square error (MSE), between the experimental and in silico data, are presented in Table 2. The O_2_ demand and CO_2_ excretion were also considered in the model evaluation (data available in Supplementary Material S3).

The iDG1237 had the lowest MSE with respect to experimental data. Furthermore, none of the models previously published were able to calculate reaction fluxes through the CA biosynthesis and clavams pathways, under phosphate limitation. Therefore, the iDG1237 model exhibited better representation capabilities of CA producing scenarios. This is most likely due to the completeness of the metabolic network, its higher connectivity and few unrealistic loops that allow a closer representation of the metabolic events that the cell experiences under nutritional and genetic constraints.

### 3.3. S. Clavuligerus Cultivation under High and Low Shear Stress Conditions

**Figure 2:**
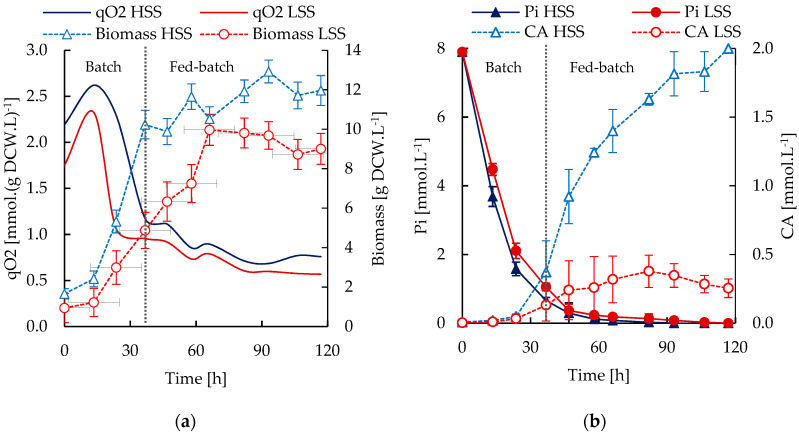

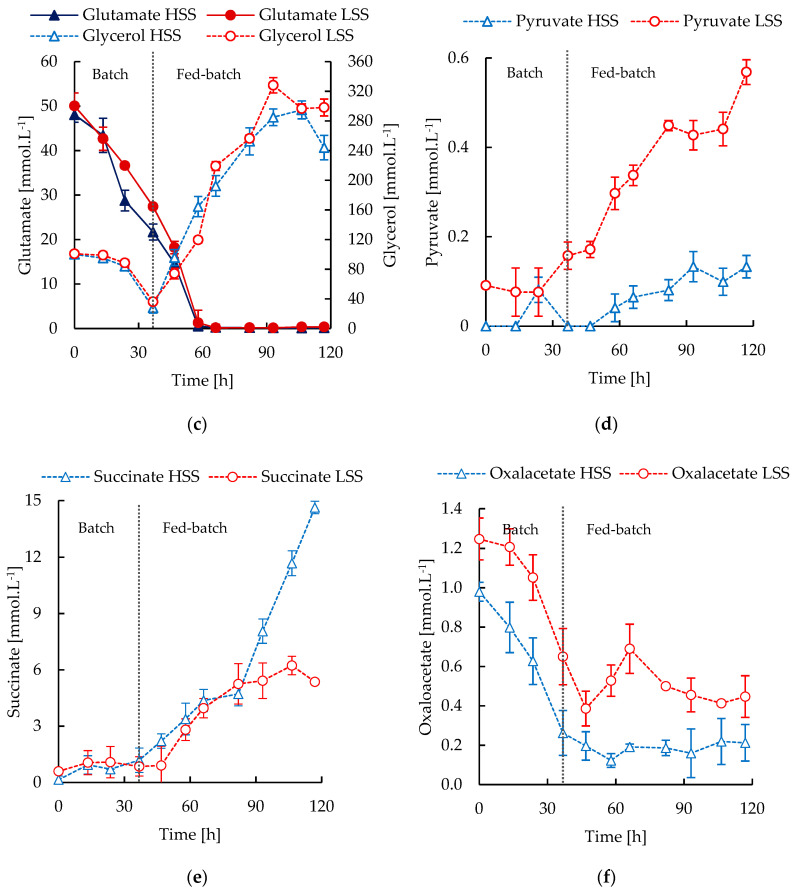

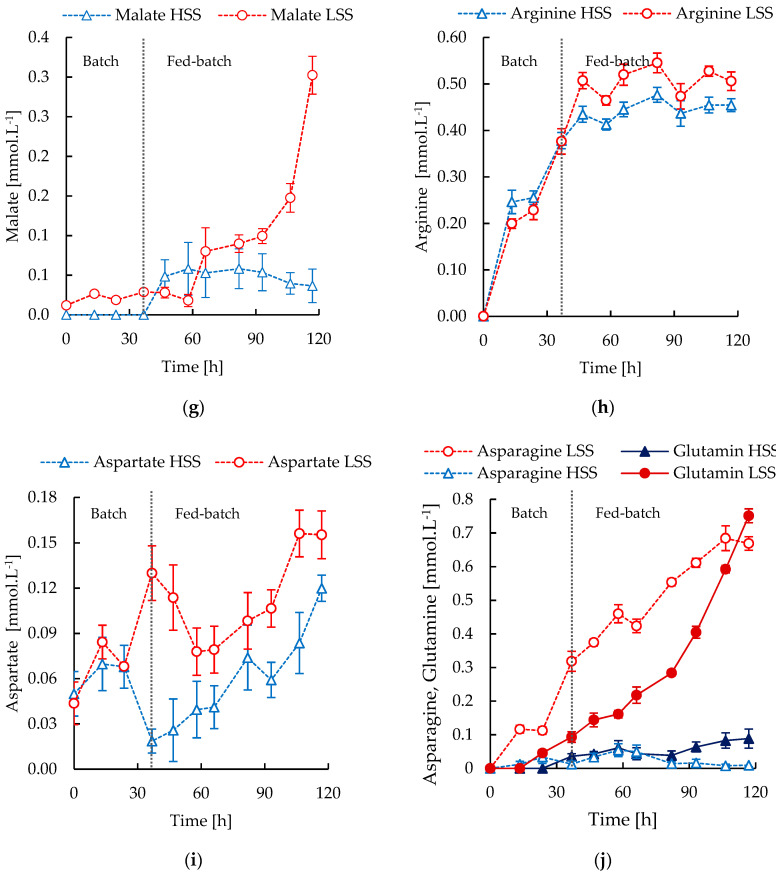
Growth and physiological profiles for fed-batch cultivations of *S. clavuligerus* under HSS (triangles, blue) and LSS (circles, red) conditions. (**a**) oxygen uptake (qO_2_) and biomass; (**b**) Phosphate (Pi) and clavulanic acid (CA); (**c**) Glutamate and glycerol; (**d**) Pyruvate; (**e**) Succinate; (**f**) Oxaloacetate; (**g**) Malate; (**h**) Arginine; (**i**) Aspartate and (**j**) Asparagine and Glutamine. HSS: High shear stress and LSS: Low shear stress.

Experimental results of growth, CA, phosphate and metabolite secretion under high shear stress (HSS) and low shear stress (LSS) conditions are shown in Figure 2. The oxygen uptake (qO_2_), growth rate and CA accumulation were higher in HSS cultivations (Figure 2a,b). The maximum growth rates of *S. clavuligerus* observed at HSS conditions were 0.104 h^−1^ and 0.028 h^−1^ for the batch and fed-batch stages, respectively. These rates were slightly higher in comparison to the 0.102 h^−1^ and 0.024 h^−1^ values attained in the LSS cultivations, during the batch and fed-batch phases, respectively. At the end of the fed-batch operation, the maximum biomass was lower in the LSS cultivations (9 g.L^−1^) than the one observed at HSS (11.9 g.L^−1^). The glycerol and glutamate concentration time courses are presented in Figure 2c. Glycerol and glutamate, as carbon sources, were consumed rapidly during the exponential growth in the batch stage. Feeding of glycerol in excess (after 37 h) led to its accumulation during the fed-batch stage. The oxygen and carbon sources uptakes were consistent with the growth rates and biomass accumulation. The maximum uptake fluxes of glycerol at HSS and LSS conditions were 1.93 and 1.92 mmol.(g DCW.h)^−1^, respectively. Similarly, the maximum uptake fluxes of glutamate were 1.34 and 1.29 mmol.(g DCW.h)^−1^ during a short time span in the batch stage.

The phosphate limitation was expected to start between 30 and 40 h and the specific CA production typically increased linked to phosphate depletion as observed in Figure 2b. The phosphate uptake was slightly lower in the LSS cultivations and therefore the phosphate exhaustion took place later (66 h) in comparison to the HSS cultivations (47 h). Even if equivalent conditions of phosphate depletion and glycerol availability were reached in both reactors, the CA production did not increase significantly under LSS conditions beyond 37 h. It was previously reported that LSS promoted mycelia thickening and branching in *S. clavuligerus*, potentially limiting the oxygen diffusion from the media to the intracellular environment [7]. In LSS cultivations, CA production was not totally inhibited, but its production was lower than the dilution and degradation rates. In contrast, under HSS, CA was continually produced above dilution caused by the feeding. This possibly favored a better diffusion of oxygen into the mycelia, that were considerably thinner and looser than the obtained in LSS cultivations. The effect of oxygen limitation on cell metabolic performance cannot be seen without a closer view of carbon flux at metabolic network level. Indeed, oxygen transfer rates remain the same under HSS and LSS conditions, providing an apparent well oxygenated cell culture environment but hampering the possible concentration gradients that might exist at microscopic level, i.e., in the proximity of the cell surface, mainly under LSS conditions. In this panorama, a much denser mycelial spatial structure may appear thus providing a lower surface to volume ratio. Moreover, under LSS, small pellets might be formed that further limit oxygen transfer at the cell–liquid media interface. Molecular oxygen has an important role in CA biosynthesis; previous reports have shown that CA production can be abolished under oxygen limitation due to a shortage of TCA-derived precursors [27]. When intracellular oxygen availability is low, oxygen is rather used for central metabolism and biomass synthesis than for secondary metabolism. CA biosynthesis increases oxygen demand and if this demand is not satisfied, the reaction fluxes along this pathway decrease as observed in LSS cultivation conditions.

Organic acid secretion in streptomycetes (Figure 2b–g) is related to an imbalance in carbon fluxes between glycolysis and the TCA cycle under carbon source abundance, also referred to as overflow metabolism [45,46]. Acid secretion can be also related with the activation of secondary metabolism in some *Streptomyces* species like *S. coelicolor* and *S. clavuligerus* [10,47]. In this regard, the higher pyruvate secretion in LSS conditions restricted the carbon flux into the TCA. Thus, pyruvate suffers a more complete oxidation leading to higher accumulation of malate and oxaloacetate in LSS than in HSS conditions. The lower repiratory quotient (RQ) registered in HSS cultivations suggest a higher imbalance and overflow metabolism. This is a consequence of higher glycerol and oxygen uptake rates, under lower mass transfer resistance derived from thinner and looser mycelia. The oxygen-dependent steps in CA biosynthesis, catalyzed by CAS, oxidizes 2-oxoglutarate to succinate. This could account for the higher accumulation of succinate in HSS cultivations, coinciding with high CA productivity, as observed in Figure 2b,e. This is also consistent with the decreasing trend and further stabilization of oxaloacetate concentration (Figure 2f), which is consumed to produce 2-oxoglutarate.

L-arginine (Figure 2h) is derived from L-ornithine, a non-proteinogenic amino acid, whose synthesis is induced under conditions of phosphate limitation [48]. HSS conditions promote a more active growth and higher nutrient uptake, leading to an earlier and more severe phosphate limitation, increasing the availability of L-ornithine and thus, L-arginine. The increased availability of GAP and L-arginine in HSS conditions led to a CA concentration almost 6-fold higher than the observed in LSS conditions. In contrast, the high pyruvate accumulation in LSS cultivation (Figure 2d) is a consequence of the decreased oxidative activity of the TCA cycle due to low intracellular oxygen availability linked to mycelia thickening.

During growth, some accumulation of oxaloacetate is expected (Figure 2f) due to the TCA cycle activity. The accumulation of oxaloacetate was higher at low CA secretion rates under LSS after glutamate exhaustion linked to the activity of the phosphoenolpyruvate carboxylase (PPC) reaction. The higher accumulation of oxaloacetate suggests a low demand of this precursor, required in the urea cycle and amino acids metabolism. The higher accumulation of L-aspartate, L-asparagine, L-arginine, and L-glutamine (Figure 2h–j) along with the lower growth rate observed in LSS conditions are indicators of lower anabolic and secondary metabolism activities. Glutamate fuels the urea cycle as a C-5 precursor yielding L-aspartate and hence arginine, which is the second early precursor of CA. Under glutamate limitation (t > 58 h), a pool of L-aspartate is expected to be formed from fumarate and ammonium, since the cultivations were not nitrogen-limited. In this scenario, the demand for glutamate and L-aspartate might also be satisfied to a lesser extent from L-glutamine and L-asparagine.

### 3.4. In Silico Metabolic Correlations of Environmental Cultivation Conditions and CA Biosynthesis

Based on the experimental results for *S. clavuligerus* cultivations in HSS and LSS conditions, six metabolic scenarios corresponding to different phases observed during the cultivation were considered for the in silico metabolic flux analysis. The complete results of the FBA simulations are available in the Supplementary Material S4. The selected scenarios coincide with different nutritional and environmental conditions attained from the bioreactor cultivations, aiming at providing insights regarding the effect of oxygen along the metabolic network in connection with the CA biosynthesis and secretion.

Experimental results showed that growth, nutrient uptake and metabolite secretion were essentially the same in both bioreactors, during the first 37 h of cultivation. Two different growth scenarios (SC1 and SC2) were observed in absence of nutrient limitation during the initial batch phase (0–37 h). From 37 h onwards, the effect of nutritional and environmental conditions on growth rate and CA biosynthesis was more pronounced. Then, two metabolic scenarios (SC3 and SC5) observed under HSS conditions, and two under LSS (SC4 and SC6) were retained as representative of the fed-batch cultivation stages covering the time interval 37–93 h. SC3 and SC4 represent growth in the presence of high glycerol and low glutamate availabilities under phosphate limitation and different RQ, as observed in the HSS and LSS cultivations. SC5 and SC6 simulate the growth on glycerol after glutamate and phosphate depletions at different RQ values. Although extracellular concentrations of phosphate and its uptake are never strictly zero given the utilization of phosphate storages during phosphate scarcity, the scenarios of phosphate limitation are characterized by a very low phosphate uptake that likely approaches to zero and consequently decreases the growth rate. Thus, scenarios of phosphate limitation SC3–SC6 were simulated by approximating the exchange flux to zero.

The scenarios with RQ = 1 (SC 4 and SC6) were observed under LSS conditions linked to lower oxygen uptake compared to carbon dioxide generation. The lower and upper bounds of the exchange fluxes for medium components were constrained between −1 and 1, respectively. The main nutrient uptakes, i.e., glycerol, glutamate, phosphate and ammonium, were set as equality constraints according to the experimentally observed fluxes, since these are the main exchange fluxes in the network. In the case of the simulations with RQ = 1, the carbon dioxide exchange flux was also constrained to satisfy the RQ constraint. The constraints of exchange fluxes for glycerol, glutamate, ammonium and phosphate, as well as simulation results for growth rate, CA and oxygen exchange fluxes are summarized in Table 3. Details of in silico medium constraints are presented in Supplementary Material S3.

The simulation of SC1 showed that flux ratio between the second phase of the glycolysis and first oxidation in the TCA cycle was close to 1.0, indicating a high activity of the TCA cycle and balanced oxidative metabolism. This is consistent with the high nitrogen and carbon uptakes during the early stages of cultivation. In those scenarios, the oxidative direction of TCA cycle was preferred to promote anabolism and growth. FBA indicates that anaplerotic reactions catalyzed by PPC, citrate lyase (CITL) and the glyoxylate shunt are not activated under such conditions.

As expected, SC2 reflects the highest metabolic activity and growth rates in silico, which is consistent with the experimental results. The experimentally verified increase of glycerol and ammonium uptakes, used as constraints in silico, leads to an RQ of 1.2 due to the increase in the CO_2_ generation. Previous reports suggested that RQ values between 1 and 1.2 are characteristic of growth without nutrient limitation, while values out of this range are associated to nitrogen, oxygen or phosphate limitations affecting also the growth rate [49,50]. Anabolism is favored by the significant carbon uptake, increasing the fluxes in the urea cycle towards L-aspartate, L-arginine, L-ornithine, and glycine synthesis, thus favoring the high biomass synthesis rate. The flux ratio between the carbon uptake and oxidation in the TCA cycle was 0.95, indicating slightly higher fluxes through the TCA cycle in the oxidative direction and increasing the yields of reduced cofactors, ATP and intermediates required by biomass synthesis. Under these conditions of non-limitation of phosphate, the flux to secondary metabolism was not favored, as experimentally observed (Figure 2a,b). Therefore, activation of numerous reactions associated to membrane lipids, nucleotides and amino acid biosynthesis was observed in addition to the highest activity, predicted in silico, through the respiratory chain (9.5 mmol.(g DCW.h)^−1^).

CA production varies because of a nutrient-specific effect in each metabolic scenario. The highest reaction flux towards the CA pathway (0.03 mmol (g DCW.h)^−1^) was observed in SC3, that is, growth under phosphate limitation and low glutamate uptake. This scenario was macroscopically characterized by a decrease in growth rate due to the low phosphate availability and hence, lower carbon and oxygen uptakes, causing a decline in the RQ down to 0.94 [50]. Oxygen uptake has an important influence on carbon flux distribution along the central carbon metabolism. Under identical scenarios of phosphate limitation (SC3 and SC4), significant differences in reaction fluxes along the TCA and urea cycles were observed as a consequence of the different oxygen uptake. Decrease in oxygen uptake, characteristic of SC4, halves the carbon fluxes in the TCA and urea cycles, reducing the flux in CA biosynthesis by a factor of 3.3, whereas carbon flux in glycolysis was slightly enhanced. These in silico results suggested a possible slowing down of TCA due to reduced oxygen availability. Slowdown of TCA in LSS cultivations would cause high pyruvate accumulation.

The calculated fluxes of reactions dependent on glutamate and related to the urea cycle in SC3 were up to 1.7-fold higher than those calculated for SC4. The carbon flux towards asparagine synthase (ASNS) decreased as consequence of the high fluxes in L-aspartate and L-glutamine synthesis via the urea cycle. These FBA results are consistent with the experimentally observed low accumulations of L-arginine, L-aspartate, L-glutamine and L-asparagine under HSS conditions in stirred tank bioreactor (Figure 2h–j), since the demand for these amino acids in a high oxygen scenario likely prevents the accumulation of these metabolites in contrast to the observations in LSS cultivations.

**Figure 3:**
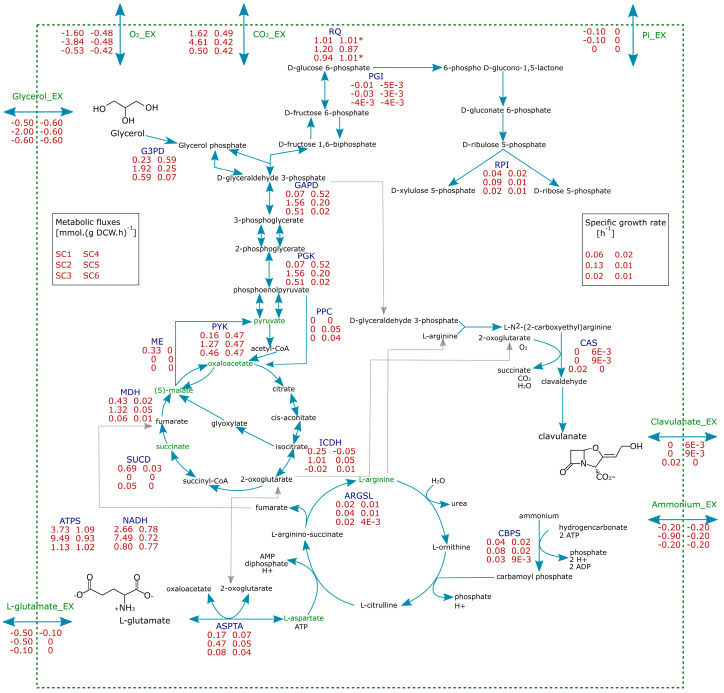
Summary of in silico carbon flux distribution throughout central metabolism and CA biosynthesis for *S clavuligerus* for the metabolic scenarios SC1–SC6. Substances in green were assayed and/or accumulated during the HSS and LSS cultivations.

In scenarios of growth under glutamate and phosphate depletions (SC5 and SC6), the flux decrease in TCA and urea cycles was higher in the oxygen restricted scenario (SC6). In contrast, in SC3 and SC4 scenarios the constrained exchange flux of L-glutamate fueled the fluxes in the urea cycle. In SC5 and SC6 these fluxes were more dependent on glycolysis and TCA, leading to a drop in the fluxes of amino acids metabolism and hence, the CA pathway. Thus, in the oxygen constrained scenarios (SC4 and SC6), the slowdown of TCA and urea cycles, as well as amino acid metabolism and CA biosynthesis, predicted in silico, suggested that intracellular limitation of oxygen in LSS cultivations causes a general drop in fluxes along central metabolism, which correlates with the experimentally observed accumulation of pyruvate, oxaloacetate, malate, and amino acids. Moreover, as a physiological objective of growth, the use of oxygen is prioritized to sustain biomass synthesis and anabolism rather than specialized metabolite synthesis. The main carbon fluxes along central metabolism are summarized in Figure 3. FVA solution ranges for representative enzymes of central metabolism are shown in Supplementary Materials S4 and S5.

Some differences between phosphate limited and non-limited scenarios were predicted in silico. High phosphate conditions repress both, CA and cephamycin C biosynthesis, in *S. clavuligerus* [51]. Since phosphate limitation affects the ATP availability, antibiotics synthesis would contribute to regulate cellular energy by supplying phosphate and other nutrients via autophagy (Type I), reduction of ATP generation (Type II) and ATP saving (Type III) [52]. They would also contribute as a metabolic sink, consuming metabolites and reducing power and ATP when the generation of the latter by catabolism exceeds the needs of anabolism in conditions of slow growth or no growth.

In the case of SC3 and SC4 scenarios, the biosynthesis of specialized metabolites might constitute a metabolic sink playing a role in growth regulation under high carbon and nitrogen availability, coexisting with a phosphate limitation. FBA simulations of the SC3 scenario demonstrated that, besides stimulation of TCA and CA biosynthesis, fluxes through ATP synthase and NADH dehydrogenase also increase compared to the oxygen constrained scenario SC4. Interestingly, the lowest fluxes through ATP synthase and NADH dehydrogenase were observed in silico in the scenario of glutamate and phosphate exhaustion (SC5), coinciding with moderate CA production in HSS conditions. In contrast, SC6 did not show reaction fluxes toward the CA pathway while the fluxes of respiratory chain dehydrogenases and ATP production were almost 10% higher than in SC5. Under these conditions CA biosynthesis seems to have a similar metabolic scenario to the synthesis of Type II antibiotics. CA biosynthesis (Figure 1) involves three dioxygen dependent oxidations, from 2-oxogluratate to succinate, and a NADPH-dependent reduction of clavulanate 9-aldehyde to clavulanic acid, transferring electrons that would likely be used in the respiration chain. Thus, CA production decreases carbon flux towards the respiratory chain, reducing the ATP generation, even if oxygen uptake is not constrained.

In silico, FBA showed that phosphate limitation causes a stoichiometric increase in the reaction fluxes in several glutamate-dependent reactions, contributing to supply fluxes of Pi and/or PPi to other reactions in the network. Additionally, the reactions in the CA pathway catalyzed by CEAS and BLS involve Pi and PPi as by-products. PPi is further hydrolyzed by the inorganic diphosphatase (PPA), whose flux increases up to 43% under P-limitation in silico. FBA results suggest that L-glutamate availability promotes the carbon flux towards CA biosynthesis in a scenario of high oxygen uptake, since L-arginine as C-5 precursor of CA is derived from L-glutamate via the urea cycle. Indeed, the highest biosynthesis rate of CA was observed in the HSS cultivations when phosphate limitation and glutamate availability coexisted with high oxygen uptake.

Furthermore, phosphate limitation is known to trigger important adaptive responses that contribute to maintain the phosphate supply to the cells in phosphate limited scenarios. Cell growth in absence or at very low concentrations of extracellular phosphate proceeds via reutilization of intracellular phosphate. Under phosphate deficiency, mother and daughter cells share intracellular resources of phosphate from RNA, polyphosphate stores, phospholipids and teichoic acids accumulated during fast growth under high phosphate availability. During the fed-batch phase with phosphate limitation (from 60 h onwards), cells do not need these constituents and are subjected to self-degradation by means of nucleases, phosphatases and related hydrolytic enzymes, releasing inorganic phosphate, pyrophosphate, and P-containing monomers into the cytoplasm. This response is controlled by the two-component system PhoR/PhoP, which is able to induce the high-affinity phosphate transport system, phosphatases, down-regulation of phosphate consuming processes and upregulation of genes involving phosphate mediated reactions [53,54]. In phosphate limited conditions, the homeostatic processes triggered to compensate the ATP deficit, lead to a strong activation of the oxidative and amino acids metabolism, resulting in abundant generation of reduced cofactors and ATP, favoring the antibiotic synthesis as a feasible mechanism to adjust the ATP generation to low phosphate availability [55,56]. Additionally, penicillin and cephalosporin antibiotics induce an ATP-consuming futile cycle of polymerization/degradation of the cell wall, regulating the ATP levels and phosphate content [52,57]. Cephamycin C secreted in low amounts by *S. clavuligerus* might induce this kind of cycle. The toxic impact of cephamycin C on the energetic metabolism could be counteracted by the action of a β-lactamase. Simultaneously, the β-lactamase levels would be regulated by CA as a β-lactamase inhibitor. These three systems with opposite effects would contribute to tightly regulate intracellular levels of CA and cephamycin C and, indirectly, the energetic metabolism of the bacteria. Thus, the high antibiotic synthesis rate observed in HSS cultivations is consistent with the high ATP generation calculated in silico in conditions of high intracellular oxygen and nutrients availability.

## 4. Conclusions

In this work, we generated an improved genome-scale metabolic model of *S. clavuligerus,* using a state-of-the-art top-down approach during the network reconstruction. The resultant curated iDG1237 metabolic model showed better predictive capabilities compared with its predecessors in relation to biomass and product biosynthesis, network topology, thermodynamic consistency and simulation of metabolic scenarios of nutritional limitation. The present model constitutes a new basis for computer-aided analysis and design of CA production scenarios as well as strain engineering of *S. clavuligerus*.

The in silico and experimental results of this study provide insights regarding the nutritional regulation of CA biosynthesis and the effects of intracellular oxygen availability in *S. clavuligerus* metabolism, when cultivated at high and low shear stress conditions. Under identical scenarios of phosphate limitation, the decrease of oxygen uptake halved the carbon fluxes in TCA and urea cycles negatively impacting CA biosynthesis. Low oxygen availability in low shear stress conditions likely leads to a slowdown of TCA cycle causing overflow metabolism (secretion of acids of the acetyl-CoA node) and accumulation of TCA intermediates due to reduction of anabolism and inhibition of antibiotic production. Experimental evidence showed that the stoichiometric effect of phosphate depletion alone does not enhance CA production, since oxygen diffusion determines the activity of oxidative and energetic metabolism. In this regard, antibiotic biosynthesis might have an important role in regulating the energetic balance of the bacteria under phosphate limitation.

The present combined experimental and modelling approach highlighted the importance of the metabolic relationship between the TCA cycle, amino acid biosynthesis and oxygen uptake under phosphate limitation during antibiotic production in *S. clavuligerus.* Further exploration of metabolic scenarios considering nutritional and genetic regulation of metabolism could contribute to the design of suitable conditions to optimize CA production and construct genetically engineered CA-overproducing strains.

## Figures and Tables

**Table 1:** Model consistency statistics of *S. clavuligerus* genome-scale metabolic models.

Feature	iMM865	iLT1021	iGG1534	iDG1237
Metabolites	1173	1162	1199	1518
Reactions	1492	1494	1534	2177
Genes	864	1021	871	1237
Reversible reactions	1492	576	610	707
Dead-end metabolites	311	333	338	48
Coupled reactions	0	422	338	880
Inconsistent coupling	0	127	35	0
Zero-flux reactions	383	473	486	105
Unbounded reactions	1024	496	598	86
TICs	411	135	185	9

**Table 2:** Comparison of experimental and in silico growth rates for *S. clavuligerus* models.

Constraints		iMM865		iLT1021		iGG1534		iDG1237		**μ_exp_**	Reference
*v*_ex,Pi_	*v*_ex,Glyc_	μ	***v*** **_ex,CA_**	μ	***v*** **_ex,CA_**	μ	***v*** **_ex,CA_**	μ	***v*** **_ex,CA_**		
-0.20	-0.50	1.60	0	0.03	0	0.92	0	0.03	0	0.04	[27]
-0.20	-0.60	1.61	0	0.04	0	0.93	0	0.04	0	0.05	[27]
-0.20	-0.93	1.67	0	0.06	0	0.97	0	0.05	0	0.07	[27]
-0.20	-2.18	2.44	0	0.12	0	1.55	0	0.09	0	0.09	[27]
0	-0.72	2.97	0	0.05	0	1.94	0	0.05	0.03	0.05	[10]
0	-1.11	2.90	0	0.05	0	1.90	0	0.05	0.04	0.05	[10]
0	-0.97	2.75	0	0.05	0	1.78	0	0.05	0.03	0.04	[10]
MSE		1.506		0.393		0.700		0.172			

*v* in [mmol. (g dry cell weight (DCW).h)^−1^], D and μ in [h^−1^].

**Table 3:** Summary of experimental constraints and in silico growth rate, CA secretion and oxygen uptake using the iDG1237 model.

Time (h)	Scenario	Shear Condition	Experimental Constraints				Results			
			*v*_ex,Glyc_	*v*_ex,Pi_	*v*_ex,NH4_	*v*_ex,Glu_	μ	*v*_ex,CA_	*v*_ex,O2_	RQ *
0–22	SC1	HSS & LSS	−0.5	−0.1	−0.2	−0.5	0.06	0	−1.6	1.01
22–37	SC2	HSS & LSS	−2.0	−0.1	−0.9	−0.5	0.13	0	−3.84	1.20
37–68	SC3	HSS	−0.6	0	−0.2	−0.1	0.02	0.02	−0.53	0.94
37–68	SC4	LSS	−0.6	0	−0.2	−0.1	0.02	6 × 10^−3^	−0.48	1.01 *
68–93	SC5	HSS	−0.6	0	−0.2	0	0.01	9 × 10^−3^	−0.48	0.87
68–93	SC6	LSS	−0.6	0	−0.2	0	0.01	0	−0.42	1.01 *
	MSE						1.8 × 10^−4^	9.8 × 10^−5^	0.28	0.02

* v in [mmol (g DCW.h)^−1^] and μ in [h^−1^]. RQ was constrained in scenarios SC4 and SC6 by matching the exchange fluxes of CO_2_ and O_2_.

## Supplementary Materials

The following are available online at https://www.mdpi.com/2076-2607/8/9/1255/s1, File S1: TICs identification scripts, File S2: Genome-scale metabolic network iDG1237 in SBML and MS Excel formats; Memote model consistency report, File S3: Model validation data-set and simulation constraints in MS Excel format, File S4: FBA and FVA simulation results for metabolic scenarios S1–S6 in MS format, File S5: Supplementary figure with FVA solution range for representative enzymes of central metabolism.
